# Effect of Traditional Chinese Medicine Combined with Bladder Perfusion with Hydroxycamptothecin on Color Ultrasound and Clinical Efficacy in Patients with Bladder Cancer Surgery

**DOI:** 10.1155/2021/7178414

**Published:** 2021-11-09

**Authors:** Shisheng Zhou, Xia Fan, Xiaojie Du, Shuang Liu, Hongmei Sun, Yan Zhang

**Affiliations:** ^1^Department of Ultrasound, Yantaishan Hospital, Yantai 264000, China; ^2^Equipment Department, Rizhao Hospital of TCM, Rizhao 76800, China; ^3^Outpatient Department, Affiliated Qingdao Central Hospital, Qingdao University, Qingdao 266042, China; ^4^Operation Room, Zhangqiu Maternity and Child Care Hospital, Jinan 250200, China; ^5^Hospital Infection-Control Department, Zhangqiu District People's Hospital, Jinan 250200, China; ^6^Department of Nutriology, Qingdao Hospital of Traditional Chinese Medicine, Qingdao Hiser Hospital, Qingdao 266033, China

## Abstract

**Objective:**

To observe the clinical effect of Xiaozheng Decoction combined with bladder perfusion with hydroxycamptothecin in the treatment of bladder cancer.

**Methods:**

A total of 92 bladder cancer patients admitted to our hospital from January to December 2018 were selected and divided into an observation group and a control group according to the random number table method, with 46 cases in each group. The observation group was given Xiaozheng Decoction combined with bladder perfusion with hydroxycamptothecin, and the control group was given hydroxycamptothecin. The levels of serum-related factors (intercellular adhesion molecule-1 (ICAM-1), E-cadherin, cell adhesion molecules (CAM), fibroblast growth factor (FGF), and vascular endothelial growth factor (VEGF)), white blood cell (WBC) level, immune function indexes, short-term total response rate, and incidence of adverse reactions were compared between the two groups before and after treatment.

**Results:**

After 2 years of postoperative treatment, the levels of ICAM-1, E-cadherin, CAM-1, FGF, and VEGF (a, b, c) in both groups were improved compared with those before treatment and the observation group was better than the control group (*p* < 0.01). The number of WBCs was significantly higher than in the control group after Traditional Chinese Medicine (TCM) treatment. The observation group was better than the control group in increasing CD3+ and CD4+ levels and decreasing CD8+ level (*p* < 0.05), indicating that this prescription could improve the immune function of patients. The recurrence rate in the observation group was 6.52% after 2 years of treatment, lower than 17.39% in the control group. Color ultrasound parameters showed that there were no statistically significant differences in arrive time (AT) and time to peak (TTP) between patients with and without recurrence and peak intensity (PI) and washout time (WT) were higher in patients with recurrence than in patients without recurrence (*p* < 0.01). The incidence of adverse reactions was significantly lower than that of the control group (*p* < 0.01).

**Conclusion:**

The clinical effect of Xiaozheng Decoction combined with hydroxycamptothecin on the treatment of bladder cancer was clear and superior to that of hydroxycamptothecin, which could effectively improve the serological indicators of patients with a low incidence of adverse reactions and prolong the survival cycle of patients. Therefore, it is worthy of promotion and application.

## 1. Introduction

Bladder cancer is the most common tumor of the urinary system in China [1], and its morbidity and mortality have shown an increasing trend in recent years [2]. Currently, transurethral bladder tumor resection (TUR-BT) is the main treatment method for nonmuscular invasive bladder cancer [3, 4]. The surgical technique is mature, and the trauma is small, but still 10%–67% of patients will relapse within 12 months. The recurrence rate is as high as 50%–70% [5–9]. Therefore, it is urgent to explore a safe and effective treatment to prevent postoperative recurrence of bladder cancer, which is an urgent topic in this field.

At present, bladder infusion of chemotherapy drugs is still the most commonly used method to prevent postoperative recurrence of TUR-BT [10–12]. There are many chemotherapeutic drugs in bladder infusion, including Bacillus Calmette–Guerin (BCG) vaccine, mitomycin, and hydroxycamptothecin [13, 14]. The recurrence rate of bladder cancer treated with BCG and mitomycin was 24.24% and 27.27%, respectively [9]. However, long-term chemotherapeutic drugs often cause chemical cystitis, bone marrow suppression, bladder contracture, and other complications [15]. Therefore, there is an urgent need for a safe and effective treatment to prevent postoperative recurrence of bladder cancer.

Any chemotherapy drugs will inevitably cause adverse reactions such as frequent urination, urgent urination, gross hematuria, low fever, and loss of appetite. Traditional Chinese Medicine (TCM) has the effect of reducing toxicity and increasing efficiency, reducing the toxic and side effects of chemotherapy drugs, improving the quality of life of patients, and playing an increasingly important role in the treatment of bladder cancer [16, 17]. TCM believes that the pathogenesis of postoperative recurrence of bladder cancer is always characterized by the deficiency of essential qi and the interaction of dampness and toxin [18, 19]. Therefore, it is suggested that supplementing qi, invigorating dampness, and detoxifying are the basic treatment methods for postoperative recurrence of bladder cancer. On this basis, Xiaozheng Decoction was used to treat postoperative recurrence of bladder cancer.

Color ultrasound examination can be used for early screening of patients with bladder cancer and follow-up observation of patients with bladder cancer. Color ultrasound examination of the urinary system has been widely used in clinical practice because of its advantages of safety, efficiency, noninvasiveness, and low cost. Color ultrasound examination of the bladder can be done by transabdominal ultrasound and transrectal ultrasound. Because of its simple and effective characteristics, it has become an important detection method for bladder cancer after surgery and can accurately determine the recurrence of postoperative tumor, which has an important reference value for the postoperative examination and diagnosis of bladder cancer.

This study was aimed to observe the clinical effect of Xiaozheng Decoction combined with bladder perfusion with hydroxycamptothecin in the treatment of bladder cancer, and results showed that the combination of hydroxycamptothecin and Xiaozheng Decoction could significantly reduce the incidence of side effects such as frequency of urination, urgency of urination, and nausea after bladder perfusion, and improve the quality of life of the patients.

## 2. Methods

### 2.1. Data and Methods

#### 2.1.1. Clinical Data

A total of 92 patients with bladder cancer admitted to our hospital from January to December 2018 were selected, including 73 males and 19 females. The average age was 65.72 ± 12.24 years. All cases were confirmed by pathological examination as transitional epithelial carcinoma. According to the classification standards of bladder cancer diagnosis and treatment guidelines in the 2014 Edition of “Guidelines for the Diagnosis and Treatment of Urological Diseases,” postoperative pathological stages of tumor were 38 cases of PTa stage, 52 cases of PT1 stage, and 2 cases of PTis stage. Pathological grades of tumor were as follows: G1, 43 cases; G2, 47 cases; and G3, 2 cases. According to the random number table method, the patients were divided into observation group and control group, 46 cases in each group. The age of the observation group was 42∼79 (65.24 ± 2.13) years, and that of the control group was 39∼78 (65.45 ± 2.41) years. This study was reviewed and approved by the Medical Ethics Committee of Qingdao Hospital of Traditional Chinese Medicine, Qingdao Hiser Hospital (approval no. 2019-12-0406). The patients and their families were aware of the study content and signed informed consent.

#### 2.1.2. Inclusion and Exclusion Criteria

*(1) Inclusion Criteria.* (1) Patients in this study were clearly diagnosed with bladder cancer. (2) All patients underwent TUR-BT. (3) Patients have normal heart, lung, liver, and kidney functions. (4) They participated voluntarily and signed informed consent forms.

*(2) Exclusion Criteria.* (1) Patients have severe cardiac and renal failure. (2) Patients have coagulation dysfunction. (3) Patients have serious cardiovascular and cerebrovascular complications, contraindications of chemotherapy, and radiotherapy. (4) Patients have primary malignancies other than bladder cancer and require concurrent use of other antitumor drugs, immunomodulators, and cytokines.

### 2.2. Treatment

#### 2.2.1. Control Group (Hydroxycamptothecin Bladder Infusion Group)

Hydroxycamptothecin injection (Shenzhen Wanle Pharmaceutical Co., Ltd., approval no. 20043063) was used as a recurrence prevention infusion drug. 40 mL of normal saline was added into 40 mg of hydroxycamptothecin and injected into the bladder by catheterization. The hydroxycamptothecin was placed in the left, right, supine, and prone positions for 30 minutes each and retained for 2 hours in total. The course of treatment is as follows: once a week, a total of 8 times, and once a month thereafter, up to 2 years after surgery.

#### 2.2.2. Observation Group (Chinese Medicine Xiaozheng Decoction Combined with the Bladder Perfusion Group)

On the basis of the bladder infusion of hydroxycamptothecin, Chinese Medicine Xiaozheng Decoction was added: Huijiaren 30 g, *Astragalus membranaceus* 20 g, Huang Jing 15 g, Hedyotis wilsa 15 g, Zhu Ling 15 g, Zedoary zedoary 9 g, and Tubei 9 g. Chinese medicine decoction is 1 dose per day, and the course of treatment was 2 years after surgery.

### 2.3. Ultrasound Examination

Philip-iu22 color ultrasonic diagnostic instrument with probe frequency of 3.5 MHz was applied. Before the examination, the patient was properly filled with the bladder, and the supine position was routinely taken, or the side decubitus position or the knee bend decubitus chest position was taken if necessary, fully exposing the lower abdomen to the symphysis pubis. The probe was carried out through multisection and multiangle scanning in the bladder region, and the lesions were carefully observed. Two-dimensional ultrasound was used to observe tumor location, morphology, size, and tumor diameter (maximum diameter and minimum diameter). During the examination, the interference of the artifact in the bladder should be avoided as far as possible, and the same patient was examined by the same physician before and after chemotherapy. The examination results were compared and analyzed and then compared with the postoperative pathological results.

### 2.4. Observation Indicators

#### 2.4.1. Serum-Related Factors

The levels of serum-related factors (ICAM-1, E-cadherin, CAM, FGF, and VEGF) in the two groups were compared before and after treatment.

#### 2.4.2. Changes in Leukocyte Values

The ratio of patients with decreased white blood cell (WBC) was observed, and the efficacy was evaluated. Grade I: 3.0 × 10^9^/L ≤ WBC < 4.0 × 10^9^/L; Grade II: 2.0 × 10^9^/L ≤ WBC < 3.0 × 10^9^/L; Grade III: 1.0 × 10^9^/L ≤ WBC < 2.0 × 10^9^/L; Grade IV: WBC < 1.0 × 10^9^/L were used to evaluate the efficacy.

#### 2.4.3. VEGF Levels

Serum VEGF levels were detected by enzyme-linked immunosorbent assay (ELISA) before and 12 months after treatment.

#### 2.4.4. Indicators of Immune Function

The levels of CD3+, CD4+, CD8+, CD4+/CD8+, and NK were detected by flow cytometry before treatment and 12 months after treatment. Before and after treatment, 5 mL of fasting venous blood was taken from the patients and placed on the automatic blood centrifugation machine in our hospital for centrifugation. The speed of centrifugation was adjusted to 3000 r/min for 10 min. The supernatant was taken and placed in a refrigerator at −20°C for examination. The relative indexes of T lymphocyte subsets in serum were detected by flow cytometry. T lymphocyte subsets (CD3+, CD4+, CD8+) and NK levels were detected by Beckman Coulter EpicsXL flow cytometry, and CD4+/CD8+ was calculated.

#### 2.4.5. Follow-Up Recurrence Rate

The recurrence cases were recorded at 6, 12, 18, and 24 months after the operation, and the percentage was calculated. Reexamination was performed by color ultrasound.

#### 2.4.6. Incidence of Adverse Reactions

A number of adverse reactions such as nausea and vomiting, frequent urination, urgent urination, abnormal urine routine, hematuria, and low fever were recorded during the treatment period, and the percentage was calculated.

### 2.5. Statistical Methods

Independent repetitions of experiments were 3 times. SPSS 20.0 software (IBM, NY, USA) was used for statistical analysis of the data. The measurement data were expressed as *x* ± *s*, and *t*-test was used for comparison between groups. Count data were expressed as frequency/rate (%), and *χ*2 test was used for comparison between groups. *p* < 0.05 was considered statistically significant.

## 3. Results

### 3.1. Color Ultrasound Image of a Patient with Preoperative Bladder Cancer

The staging of bladder tumors was based on the TNM staging method (6^th^ edition, 2002) of the International Union against Cancer: Stage Ta: noninvasive, superficial tumor; T1: tumor invasion into subepithelial connective tissue; Stage T2a: tumor invasion of the superficial muscle layer; Stage T2b: tumor invasion of deep muscle layer; and >Stage T2: tumor invasion of the peribladder tissue. Imaging features of bladder tumor stage were determined by 3D-CEUS: Ta stage: the bladder wall at the base of the tumor was clear, and the angiography showed continuous and bright lines of the bladder wall; T1: the contrast agent perfusion was slightly stronger in the mucosa layer of the bladder wall than in the muscle layer. T2a stage: <1/2 bladder wall and tumor contrast agent perfusion synchronous, consistent degree of enhancement; T2b stage: >1/2 bladder wall and tumor contrast agent perfusion synchronous, consistent degree of enhancement; and >Stage T2: full bladder wall or surrounding tissue perfusion time, intensity, and characteristics of bladder tumor.

Among the 46 cases of bladder cancer in the control group, 27 tumors were located in the trigone, 18 cases were located in the lateral wall, and 1 case was located at the top. Among the 46 cases of bladder cancer in the observation group, 28 cases were located in the trigone region, 17 cases were located in the lateral wall, and 1 case was located at the top. The characteristics of two-dimensional ultrasonography of patients in both groups were improved after surgery.

### 3.2. Short-Term Efficacy Evaluation

According to the Guideline for the evaluation of TCM Clinical Efficacy in bladder cancer [20], TCM curative effect of bladder cancer was defined as clinical benefit, which was divided into obvious benefit, benefit, and no benefit. A significant benefit is as follows: effective or stable tumor evaluation + significant effect of symptom evaluation + effective or stable quality of life evaluation + effective or stable body weight evaluation. Benefits are as follows: stable tumor evaluation + effective symptom evaluation + effective or stable quality of life evaluation. No benefit is as follows: those who do not meet the above targets. All patients observed in this study were post-TUR-BT patients, so tumor evaluation was not used as the criteria for clinical efficacy.

After 2 years of postoperative treatment, the total effective rate in the observation group was 91.30%, which was higher than 67.39% in the control group (*p* < 0.05), as shown in Table 1.

### 3.3. Comparison of Serum-Related Factor Levels

Before treatment, there was no significant difference in the levels of ICAM-1, E-cadherin, CAM-1, FGF, VEGF (a, b, c) between the two groups (*p* < 0.05). After 5 courses of treatment, the levels of ICAM-1, E-cadherin, CAM-1, FGF, and VEGF (a, b, c) in both groups were improved compared with before treatment, and the observation group was better than the control group (*p* < 0.01), as shown in Table 2.

### 3.4. Changes in White Blood Cell Values

Table 3 shows 11 (23.91%) patients with leukocyte suppression in the observation group and 27 (58.70%) patients in the control group. The observation group has significantly better efficacy than the control group.

**Figure 1:**
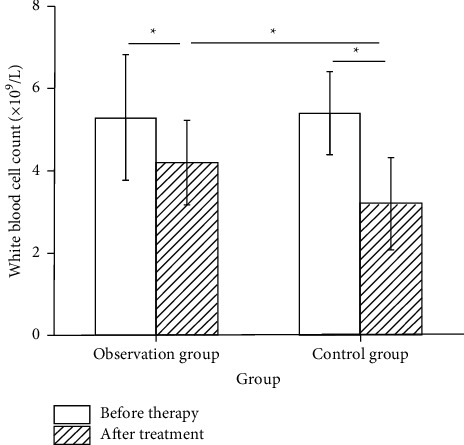
Comparison of the number of WBC between the two groups of patients before and after treatment. ^*∗*^*p* < 0.05.

According to Figure 1, there was no significant difference between the two groups before treatment (*p* > 0.05), and the internal comparison between the two groups after treatment was statistically significant (*p* < 0.05). There were still significant differences between the two groups after treatment (*p* < 0.05), indicating that the number of WBC in the observation group after treatment was significantly higher than that in the control group.

### 3.5. Comparison of Immune Function Indexes between the Two Groups before and after Treatment

**Figure 2:**
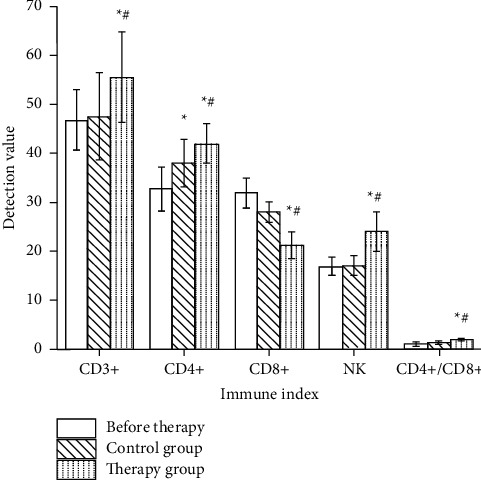
Comparison of immune function indexes between the two groups before and after treatment. ^*∗*^*p* < 0.05 versus after treatment; ^#^*p* < 0.05 versus control group.

The level of CD4+/CD8+ in the control group was significantly increased after treatment (*p* < 0.05). CD3+ and CD4+ levels were higher (*p* < 0.05) and CD8+ level were lower than those before treatment (*p* < 0.05). CD3+, CD4+, and CD4+/CD8+ levels and NK level in the observation group were significantly increased after treatment (*p* < 0.05). CD8+ level were significantly decreased (*p* < 0.05). After treatment, the increase of CD4+/CD8+ level in the observation group was significantly better than that in the control group (*p* < 0.05). After treatment, the observation group was superior to the control group in increasing the levels of CD3+, CD4+, and NK and decreasing CD8+ level (*p* < 0.05), as shown in Figure 2, suggesting that this formula can improve the immune function of patients.

### 3.6. Comparison of Serum VEGF Levels between the Two Groups before and after Treatment

There was no significant difference in serum VEGF levels between the two groups before treatment (*p* > 0.05). After three months of treatment, there was no significant difference in serum VEGF levels between the two groups (*p* > 0.05). After six and twelve months of treatment, VEGF levels in both groups were significantly decreased, and VEGF levels in the observation group and control group were significantly decreased after treatment (*p* < 0.05). Compared with the two groups after treatment, the observation group was superior to the control group in reducing VEGF level (*p* < 0.05), as shown in Table 4.

### 3.7. Recurrence Rate

**Figure 3:**
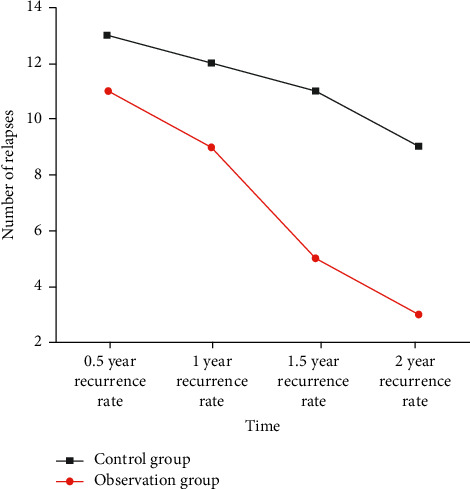
Comparison of efficacy in the recurrence rate of bladder tumor between the two groups.

All patients in this study were treated with perfusion and followed up for 24 months. Specific results: the recurrence rates of bladder tumors in the observation group were 23.91%, 17.39%, 10.86%, and 6.52% at 0.5, 1, 1.5, and 2 years, respectively, while those in the control group were 28.26%, 26.09%, 23.91%, and 17.39% at 0.5, 1, 1.5, and 2 years, respectively. The recurrence rate of bladder tumor in the observation group was 4.35%, 8.70%, 13.05%, and 10.87% lower than that in the control group, respectively. After statistical treatment, there was no significant difference in recurrence rate between the two groups at 0.5 years and 1 year (*p* > 0.05). The recurrence rate in the observation group was significantly lower than that of the control group at 1.5 and 2 years (*p* < 0.05). In conclusion, compared with the control group, the effect of the observation group on reducing the recurrence rate of bladder tumor was increasingly obvious over time, as shown in Figure 3.

### 3.8. Comparison of Parameters of Contrast-Enhanced Ultrasound in Bladder Cancer Patients with and without Recurrence at Two Years

In the observation group, 3 of the 46 patients were positive by ultrasonography, the maximum tumor size was 0.9 cm × 0.5 cm, and the minimum tumor size was 0.6 cm × 0.4 cm. The tumor was broad base, papillary, triangular, mound, or corrugated protruding into the bladder with regular or irregular protruding edges. There was no boundary or fuzzy boundary between the base edge and the bladder wall, and the internal echo was uneven low, equal, or slightly strong. The bladder wall in the tumor area had thickening, stiffness, or no hierarchical sonographic changes. The recurrence of bladder cancer was confirmed by surgical pathology.

In the observation group, preoperative ceUS showed rapid filling of contrast agent in arterial phase in the mass, with arrival time earlier than the bladder wall, prostate, or cervix, and the intensity of contrast agent filling was higher than the bladder wall, prostate, or cervix. In the delayed stage, the internal mass was gradually cleared of contrast media. There was no significant difference in AT and TTP between recurrent and nonrecurrent patients (*p* > 0.05). PI and WT in recurrent patients were higher than those in nonrecurrent patients, and the differences were statistically significant (*p* < 0.05), as shown in Table 5. In patients with recurrence, 66.7% (2/3) showed fast forward and slow regression in contrast enhancement mode, and 33.3% (1/3) showed fast forward and slow regression in pathological grading. Among the patients without recurrence, 86.0% (37/43) showed fast forward and fast regression in contrast enhancement mode, and 14.0% (6/43) showed fast forward and slow regression in pathological grading.

### 3.9. Adverse Reactions

**Figure 4:**
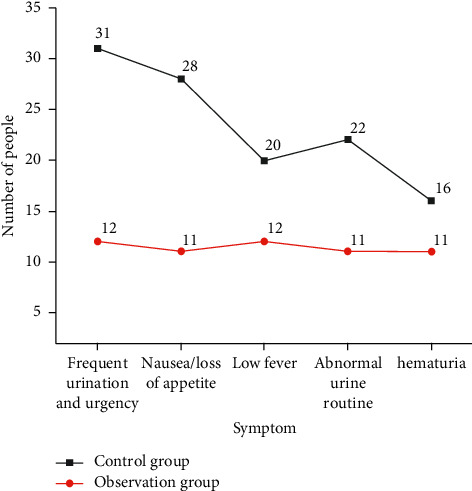
Comparison of adverse reactions between the two groups.

In the control group, 31 cases had frequent urination, 28 cases had nausea or loss of appetite, and 22 cases had abnormal urine routine. In the observation group, 12 cases of frequent urination, 11 cases of nausea or anorexia, and 11 cases of abnormal urine routine, Chinese medicine combined with hydroxycamptothecin can significantly reduce the incidence of frequent urination, urgent urination, nausea, and abnormal urine routine after bladder perfusion, and the difference was significant (*p* < 0.05). Meanwhile, there were 20 cases of low fever in the control group, 12 cases of low fever in the observation group, 16 cases of hematuria in the control group, and 11 cases of hematuria in the observation group. There was no significant difference between the two groups (*p* > 0.05). No other adverse reactions such as myelosuppression were found during the treatment (Figure 4).

## 4. Discussion

Bladder cancer is one of the most common solid tumors in the genitourinary system and the second most common malignant tumor in the genitourinary system, with more than 400,000 new cases occurring every year [21]. At present, more than 2 million people in China suffer from bladder cancer [22]. There were 430,000 new cases of bladder cancer in 2012, making it the ninth most common cancer worldwide. In recent decades, there has been little success in reducing the incidence of bladder cancer for many reasons [23]. Bladder cancer has a high postoperative recurrence rate due to being multifocal, implantable, and urinary-derived [24]. 50%∼70% of patients have recurrence after bladder-sparing surgical treatment, and 20%∼30% of superficial bladder cancer develops into invasive or metastatic cancer after surgery, with poor long-term efficacy [25, 26]. All bladder cancer patients who underwent bladder preservation had a higher risk of recurrence. Therefore, postoperative local perfusion chemotherapy should be performed to kill the remaining tumor cells as much as possible, prevent tumor progression, reduce the probability of recurrence, and delay recurrence. At present, although there are many chemotherapeutic drugs available for postoperative bladder infusion chemotherapy, about 30% of patients still have a recurrence.

Previous studies have found that hydroxycamptothecin is basically not absorbed by the bladder mucosa. It inhibits DNA replication, transcription, and mitosis by acting on DNA topoisomerase I and has an inhibitory effect on the development of tumors [27, 28]. The adverse reactions are relatively small, and the patient's recurrence rate is low, which is better than the preventive effect of mitomycin and BCG. In our hospital, hydroxycamptothecin has been used for adjuvant chemotherapy after bladder preservation in patients with bladder cancer, and good efficacy has been achieved for many years. However, because most patients are older at the onset of the disease, and the body constitution is in the decline stage, and the return of surgical treatment, the body's resistance is significantly reduced, and it can also be seen in the clinic that the patient has severely reduced white blood cells and decreased body immunity due to myelosuppressive reaction after perfusion, resulting in severe infections and a poor prognosis for the patient.

TCM can enhance the curative effect of chemotherapy drugs and the immune function of the body. This study explored the effect of hydroxycamptothecin bladder perfusion (control group) and Xiaozheng Decoction combined with hydroxycamptothecin bladder perfusion (observation group) to prevent bladder cancer recurrence after surgery. The recurrence rates at 1.5 years and 2 years after surgery were lower than those in the control group (*p* < 0.05), and with the passage of time, the recurrence rate of patients in the observation group gradually decreased.

Long-time bladder perfusion chemotherapy can cause different degrees of adverse reactions. This study showed that the adverse reactions in the treatment of the two groups were mainly manifested as anorexia, nausea and vomiting, hematuria, low fever, frequent urination and urgent urination, and abnormal urine routine. Pharmacological experiments showed that Xiaozheng Decoction can inhibit tumor formation, enhance immunity, and reduce adverse reactions caused by chemotherapy drugs. This present study indicated that Xiaozheng Decoction combined with hydroxycamptothecin bladder infusion can reduce the incidence of anorexia, nausea and vomiting, frequent and urgent urination, and abnormal urine routine and improve the quality of life of patients to a certain extent. VEGF is highly specific and can induce the proliferation of vascular endothelial cells and promote the angiogenesis of tumor cells, which plays an important role in the occurrence and development of bladder tumor and has a certain relationship with tumor recurrence [29, 30]. Therefore, the detection of VEGF level can be used to determine the status of tumor metastasis and treatment effect. In this present study, that the levels of VEGF in the two groups showed a downward trend after treatment. At 6 and 12 months after treatment, the VEGF levels in the observation group were lower than those in the control group (*p* < 0.05). These results indicated that the combination of Chinese and Western drugs can reduce the level of VEGF in postoperative patients with bladder cancer, which is beneficial to reducing the recurrence and metastasis of tumor.

The application of chemotherapeutic drugs is often accompanied by serious gastrointestinal reactions, bone marrow suppression, nerve damage, and other toxic and side effects, and many patients often end treatment halfway because they cannot tolerate the toxic and side effects of chemotherapy [31]. The results of this study showed that the white blood cells count in the observation group was significantly higher than that of the control group after TCM treatment. In addition, the observation group was superior to the control group in increasing the levels of CD3+, CD4+, and NK and decreasing the level of CD8+ (*p* < 0.05), indicating that this prescription could improve the immune function of patients. These results suggested that TCM combined with hydroxycamptothecin infusion in the treatment of bladder cancer had a significant protective effect on white blood cells, which was worthy of popularization and application.

## 5. Conclusion

In conclusion, Xiaozheng Decoction combined with hydroxycamptothecin bladder perfusion can significantly reduce the recurrence rate after TUR-BT; reduce the incidence of frequent urination, urgency, nausea, and abnormal urine routine after bladder perfusion; and improve the quality of life of patients. Therefore, the combination of TCM and Western medicine treatment can improve the clinical efficacy and alleviate the suffering of patients, which is worthy of clinical promotion. However, the mechanism of Xiaozheng Decoction remains to be further studied.

## Figures and Tables

**Table 1:** Comparison of short-term efficacy evaluation between the two groups.

Group	Complete remission	Partial remission	Stable	Progress	Total effective rate (%)
Control group	3(6.52)	13(28.26)	15(32.61)	15(32.61)	67.39
Observation group	12(26.09)	17(36.96)	16(34.78)	4(8.70)	91.30

**Table 2:** Comparison of serum-related factors between the two groups before and after treatment.

Project	Observation group		Control group	
	Before treatment	After treatment	Before treatment	After treatment
ICAM-1 (ng/L)	75.98 ± 17.11	35.03 ± 6.22^*∗*^	75.73 ± 17.88	47.14 ± 9.03^*∗*#^
E-cadherin (ng/L)	76.90 ± 13.78	37.02 ± 5.34^*∗*^	77.02 ± 14.01	64.22 ± 5.52^*∗*#^
CAM (ng/L)	21.54 ± 3.85	71.30 ± 13.64^*∗*^	21.67 ± 4.12	38.71 ± 8.09^*∗*#^
FGF (pg/L)	26.14 ± 6.72	7.73 ± 2.75^*∗*^	26.34 ± 7.00	13.66 ± 3.23^*∗*#^
VEGF-a (*μ*g/L)	196.32 ± 26.24	82.37 ± 5.07^*∗*^	197.21 ± 27.33	162.87 ± 14.28^*∗*#^
VEGF-b (*μ*g/L)	126.45 ± 16.02	105.98 ± 12.73^*∗*^	126.08 ± 16.21	65.63 ± 3.79^*∗*#^
VEGF-c (*μ*g/L)	151.35 ± 13.03	123.96 ± 7.33^*∗*^	151.12 ± 13.22	71.99 ± 6.93^*∗*#^

^*∗*^ *p* < 0.05 versus after treatment; ^#^*p* < 0.05 versus the control group.

**Table 3:** Comparison of white blood cell suppression grades between the two groups (*n* (%)).

Group	*n*	I	II	III	IV	Total
Observation group	46	8(17.39)	2(4.35)	1(2.17)	0(0.00)	11(23.91)
Control group	46	13(28.26)	8(17.39)	4(8.69)	2(4.35)	27(58.70)

**Table 4:** Comparison of serum VEGF levels between the two groups before and after treatment.

Group	Before treatment	Three months after treatment	Six months after treatment	Twelve months after treatment
Observation group	388.5 ± 98.4	346.3 ± 114.4	349.7 ± 110.8	308.2 ± 152.8
Control group	389.7 ± 99.7	378.9 ± 130.7	388.7 ± 102.6	356.3 ± 159.2
*t*	0.597	1.598	2.445	2.014
*p*	0.551	0.097	0.021^*∗*^	0.049^*∗*^

**Table 5:** Comparison of contrast ultrasonography parameters of recurrent and nonrecurrent bladder cancer patients in the observation group.

Group	Cases	AT (s)	TTP (s)	PI (dB)	WT (s)
Relapsed patients	3	21.6 ± 1.5	30.9 ± 2.4	22.0 ± 12.2	36.7 ± 10.3
Nonrelapsed patients	43	22.0 ± 1.7	29.1 ± 1.8	14.9 ± 8.9	21.9 ± 8.1

## Data Availability

The datasets used and/or analyzed during the present study are available from the corresponding author on reasonable request.
